# Supplementary material for: The Usefulness of Resistant Maltodextrin and Chitosan Oligosaccharide in Management of Gut Leakage and Microbiota in Chronic Kidney Disease
Source: Nutrients. 2023 Jul 28;15(15):3363. doi: 10.3390/nu15153363 (PMC10420640; doi:10.3390/nu15153363)
Supplement: Supplementary file 1 [file nutrients-15-03363-s001.zip › nutrients-2513913-supplementary.pdf]

Supplementary

Table S1. Estimated creatinine clearance calculated from serum creatinine by ACRALA (https://idal.uv.es/aclara/)

	Control	CKD	Inulin	COS	RMD
eClCr (mL/min)	3.80±0.42	2.24±0.97*	2.05±0.81*	2.86±0.72	2.41±1.17
(estimate) % of control		58.9%	53.9%	75.3%	63.4%

*P<0.05 compared with Control

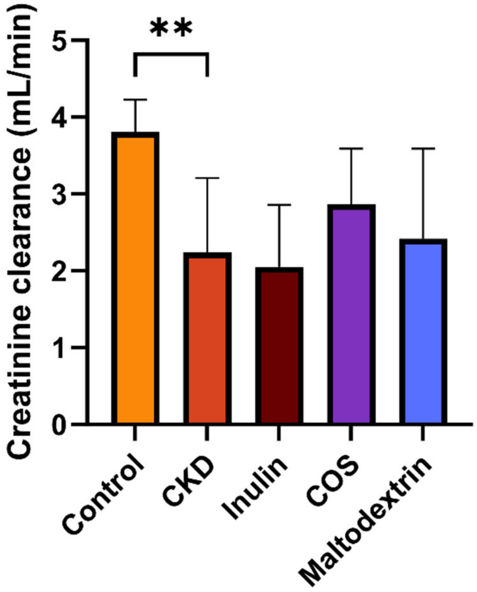

Figure S1. Estimated creatinine clearance calculated from serum creatinine by ACRALA (https://idal.uv.es/aclara/)
